# Treatment of Posterior Shoulder Instability in National Hockey League Players: A Survey of NHL Team Physicians

**DOI:** 10.1177/23259671261440208

**Published:** 2026-05-26

**Authors:** Tyler M. Hauer, Elise B.E. Raney, Ryan T. Lin, Dharmesh Vyas

**Affiliations:** † Department of Orthopaedic Surgery, UPMC Freddie Fu Sports Medicine Center, University of Pittsburgh, Pittsburgh, Pennsylvania, USA; ‡ Division of Orthopaedic Surgery, University Health Network, University of Toronto, Toronto, Ontario, Canada; § University of Pittsburgh School of Medicine, Pittsburgh, Pennsylvania, USA; ‖ Pittsburgh Penguins Performance and Sport Science, UPMC Lemieux Sports Complex, Pittsburgh, Pennsylvania, USA; Investigation performed at University of Pittsburgh Medical Center Lemieux Sports Complex, Pittsburgh, Pennsylvania, USA

**Keywords:** shoulder, instability, glenoid labrum, ice hockey

## Abstract

**Background:**

Posterior shoulder instability is a common yet underappreciated condition in elite hockey players. It presents unique treatment dilemmas during both the season and the offseason.

**Purpose:**

To investigate treatment trends and decision-making factors among National Hockey League (NHL) team physicians when managing posterior shoulder instability.

**Study Design:**

Cross-sectional study.

**Methods:**

A 35-item questionnaire addressing treatment decisions, surgical thresholds, bone loss management, and return-to-play (RTP) protocols was sent to all 32 NHL team orthopaedic surgeons. Data were collected on both in-season and offseason preferences and analyzed as descriptive statistics.

**Results:**

Of the 32 invited team physicians, a total of 31 (97%) completed all (n = 29) or most (n = 2) of the survey. For first-time in-season posterior shoulder instability, 100% of physicians chose nonoperative management. For recurrent posterior instability, 35% favored in-season surgery if there was no bone loss, and 74% favored in-season surgery when bone loss was ≥15%. Timing within the season did not influence the majority of surgeons. In the offseason, 39% would recommend offseason surgery for first-time injuries and 48% for recurrent instability without bone loss. Arthroscopic stabilization was the preferred procedure in all soft tissue cases; in cases of bone loss ≥15%, 53% would perform a soft tissue only procedure, and 30% would perform augmentation with distal tibial allograft. RTP was guided by full strength, range of motion, absence of pain, and absence of subjective instability.

**Conclusion:**

NHL team physicians strongly favor nonoperative management in-season for first-time posterior shoulder instability events. Recommendation of in-season surgery becomes more likely in the presence of injury recurrence or bone loss. Heterogeneity exists among surgeons regarding the amount of combined bone loss that would lead to the recommendation of a bony procedure. Injury timing during the season rarely alters treatment decisions, and arthroscopic repair remains the standard approach.

Posterior shoulder instability poses substantial challenges in elite contact sports. Previous studies highlight variable outcomes with both operative and nonoperative treatment, particularly in the presence of bone loss. Despite recognition of its career impact, management strategies remain heterogeneous, and no study has reported treatment strategies among National Hockey League (NHL) team physicians.

By surveying NHL team physicians, this study defines current practice trends for posterior shoulder instability in professional hockey. It demonstrates agreement on nonoperative management of first-time in-season injuries but exposes heterogeneity in surgical thresholds, bone-loss management, and return-to-play (RTP) clearance, informing future consensus-building and research.

Posterior shoulder instability represents a significant yet often underreported injury in elite collision athletes, particularly in the NHL. Its subtle presentation—often as vague pain or subclinical subluxation—contrasts with the dramatic nature of anterior instability and complicates early diagnosis and management. Decision-making in the high-stakes professional sports setting is influenced by the athlete's ability to perform at a high level, timing during the season, risk of recurrence, and implications on future joint health.

Although posterior shoulder instability accounts for a smaller proportion of overall instability cases—ranging from 2% to 18%—its incidence is growing in contact sports, particularly among athletes subjected to repetitive shear forces across the posterior capsule.^9,14^ The mechanism of injury often involves repetitive trauma with the shoulder in forward flexion and internal rotation, leading to injury of the posterior capsulolabral complex.^8^ These subtle injuries may not cause complete dislocation but can result in considerable functional impairment, including pain, instability, and diminished performance.^1,6^

Recent epidemiological studies have shown that posterior shoulder instability can have a lasting impact on performance metrics and career longevity, especially in collision sports.^11,13^ In a review of National Football League Combine participants, players with posterior labral injuries were found to be drafted later and had significantly reduced early-career performance, particularly when managed nonoperatively.^13^ Furthermore, surgical management for posterior capsulolabral injuries has shown successful outcomes, patient satisfaction, and RTP.^2,12^ Despite these insights, no formal guidelines exist for managing posterior shoulder instability in elite hockey players. Treatment decisions often depend on a combination of clinical presentation, imaging findings (eg, bone loss), and timing during the season (eg, playoffs vs. offseason).

No studies in the literature have focused solely on posterior shoulder instability in NHL athletes. The purpose of this study was to investigate and report on the treatment preferences of NHL team orthopaedic surgeons when managing posterior shoulder instability. We hypothesized that, similar to anterior instability, physicians would favor nonoperative treatment for first-time injuries during the season and opt for surgical stabilization in cases of recurrent injury or bone loss.

## Methods

We developed and administered a survey consisting of 35 sport-specific questions focusing on the preferred treatment approach for an NHL hockey player who sustained a posterior shoulder instability event. Considerations incorporated into the survey included whether the injury was a first-time or recurrent episode, at what point during the season the injury occurred (first half of the season, second half of the season with the team in playoff contention, or during the playoffs), the presence or absence of glenoid bone loss, and both the operative and the nonoperative management preferences of the participating surgeon (including postoperative rehabilitation and RTP). The survey questions were uploaded to Research Electronic Data Capture (REDCap; Vanderbilt University), a secure web-based application that is utilized for building and managing online surveys and databases. This study was determined to be exempt from institutional review board approval, and all responses were collected anonymously. In January 2025, invitations to complete the survey were electronically distributed to 32 NHL team orthopaedic surgeons (representing all 32 teams in the NHL). The respondents consisted entirely of orthopaedic surgeons who serve as team physicians for their respective organizations, and therefore, all questions regarding surgical management could be answered appropriately without consultation from other medical staff. Each invitation included a secure REDCap link and detailed instructions for completing the survey. REDCap parameters were configured to anonymize participant identities and response sets. Data collection continued through May 2025, at which point the survey was closed. All responses were compiled using Microsoft Excel, and the results were analyzed descriptively as counts and percentages.

## Results

**Figure 1.**
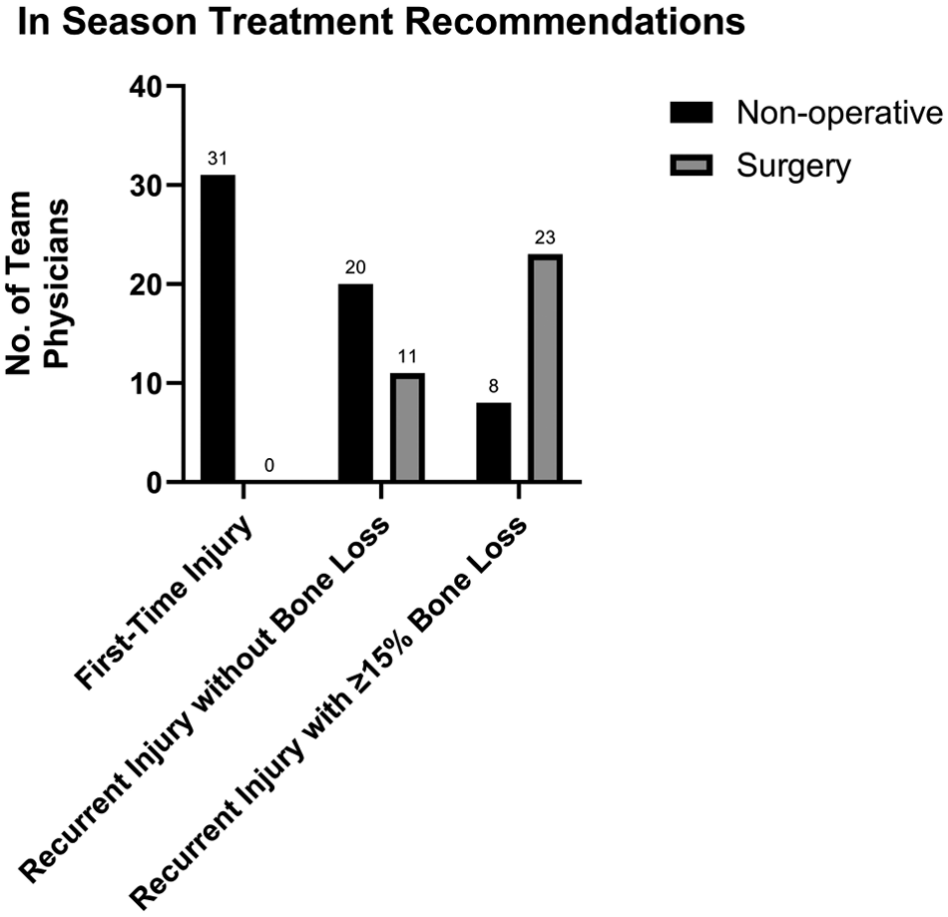
Survey results for in-season management recommendations of posterior shoulder instability.

Of the 32 NHL team orthopaedic surgeons surveyed, 31 (97%) responded to the survey. Of these 31 surveys, 29 surveys (94%) were completed in their entirety, and 2 surveys (6%) were mostly completed. Of the 2 incomplete surveys, 1 survey was missing 8 out of 35 responses (77% completion) and the other was missing 1 response (97% completion). The mean experience of the respondents was 23.8 ± 8.4 years in practice and 14.4 ± 8.4 years as an NHL team surgeon. The mean number of annual posterior shoulder stabilization procedures performed among the respondents was 21.3 (range, 0-150). Regarding in-season management, all 31 respondents (100%) preferred nonoperative treatment for first-time posterior instability injuries. For recurrent injuries without bone loss that occurred during the first half of the season, 11 respondents (35%) were likely to recommend in-season surgical management. However, for recurrent injuries, when combined bone loss (glenoid and reverse Hill-Sachs) reached or exceeded 15%, the proportion recommending in-season surgery increased to 23 respondents for injuries occurring during the first half of the season (74%) (Figure 1). The most commonly cited factors influencing in-season surgical intervention included the presence of bone loss (27/31 respondents; 87%), same-season recurrence of posterior dislocation (26/31 respondents; 84%), recurrent pain (24/31 respondents; 77%), subjective instability reported by the player (21/31 respondents; 68%), performance decline attributed to the instability (20/31 respondents; 65%), same-season recurrence of posterior subluxation (18/31 respondents; 58%), and recurrent posterior dislocation with last event in a prior season (17/31 respondents; 55%). A total of 26 respondents (84%) reported that they would be more likely to recommend in-season surgery to a player with subjective posterior instability over a player with isolated shoulder pain without subjective instability. Regarding imaging, 20 respondents (65%) reported obtaining a computed tomography scan of the injured shoulder in the setting of suspected bone loss on radiograph or magnetic resonance imaging.

When asked whether timing of the injury during the season affected treatment decisions in the setting of first-time instability, all physicians indicated it did not. When asked whether timing of the injury during the season affected treatment decisions in recurrent instability, most physicians indicated it did not. In the setting of recurrent instability without bone loss, 8 respondents (26%) felt they would be more likely to attempt nonoperative management and RTP if the injury occurred in the second half of the season with the team in playoff contention. Similarly, in the setting of recurrent instability without bone loss, 6 respondents (19%) felt they would be more likely to attempt nonoperative management and RTP if the injury occurred in the playoffs. In the setting of recurrent instability with ≥15% combined bone loss, responses were similar. Seven respondents (23%) felt they would be more likely to attempt nonoperative management and RTP if the injury occurred in the second half of the season with the team in playoff contention. Similarly, in the setting of recurrent instability with ≥15% combined bone loss, 10 respondents (32%) felt they would be more likely to attempt nonoperative management and RTP if the injury occurred in the playoffs.

**Figure 2.**
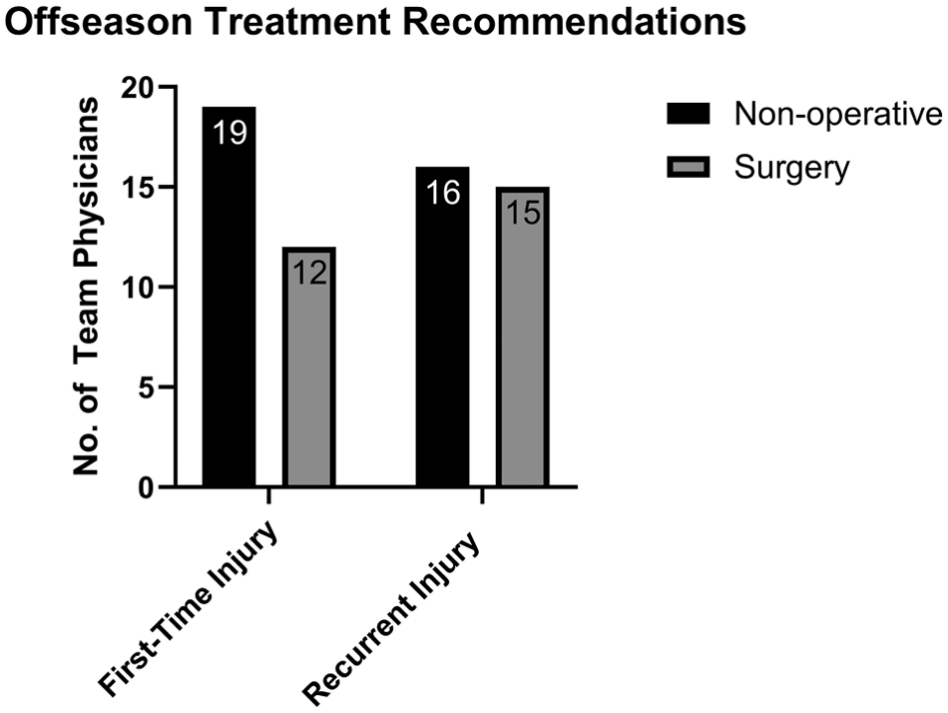
Survey results for offseason management recommendations of posterior shoulder instability.

In terms of offseason management, 12 respondents (39%) would recommend offseason surgery for a first-time posterior instability event, while 15 respondents (48%) would recommend offseason surgery for a recurrent posterior instability event in the absence of bone loss (Figure 2). The most influential factors toward recommending offseason surgery in first-time posterior instability events were persistent pain after adequate rehabilitation (31 respondents; 100%), associated pathology such as anterior labral extension of tear or partial-thickness rotator cuff tear (22 respondents; 71%), and large labral tear size (19 respondents; 61%). The most influential factors toward recommending offseason surgery in the setting of a recurrent posterior instability event were higher probability of recurrence (27 respondents; 87%), presence of bone loss (26 respondents; 84%), player preference (24 respondents; 77%), worsening labral tear extension (15 respondents; 48%), and the likelihood of further plastic deformity of the posterior capsule with each instability event (10 respondents; 32%).

Regarding surgical technique, all 31 respondents (100%) preferred arthroscopic stabilization in cases involving isolated soft tissue injury without bone loss. For cases involving ≥15% bone loss, 16 respondents (53%) favored arthroscopic stabilization without bony augmentation, 9 respondents (30%) preferred open posterior stabilization using a distal tibial allograft, 2 respondents (7%) favored open stabilization without bony augmentation, and 3 respondents (10%) stated that their approach would be case dependent. Reported thresholds for performing a bony procedure varied: 2/29 respondents (7%) indicated ≥10% bone loss as their threshold, 10 respondents (34%) indicated ≥15%, 9 respondents (31%) indicated ≥20%, and 4 respondents (14%) indicated ≥25%. Notably, 4 respondents (14%) indicated that they would never perform a bony procedure regardless of bone loss.

**Figure 3.**
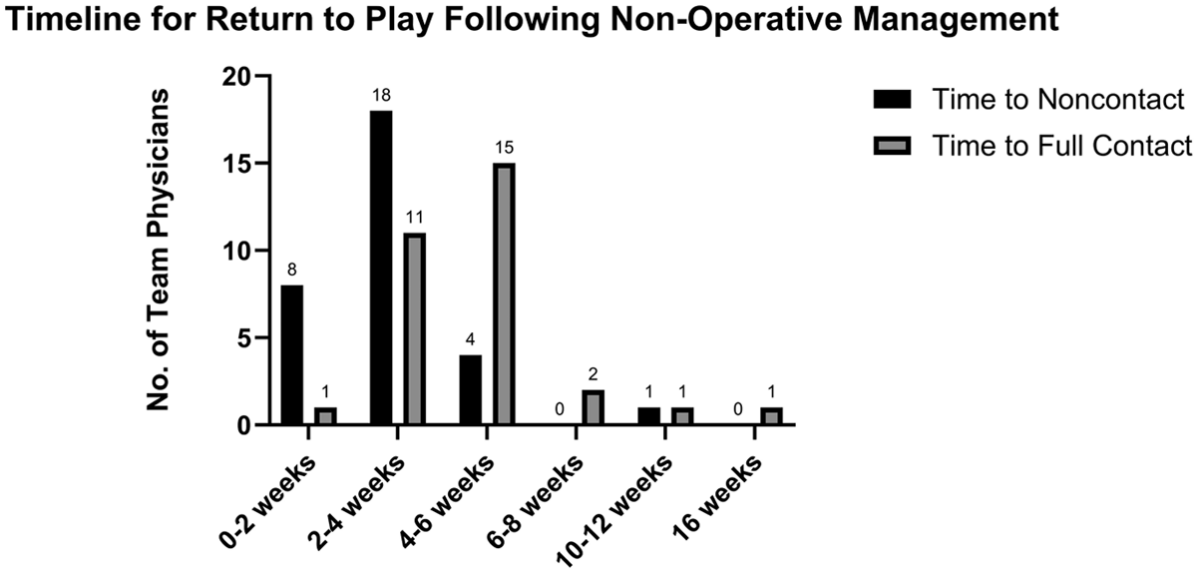
Survey results for return-to-play timeline recommendations of posterior shoulder instability following nonoperative management.

**Figure 4.**
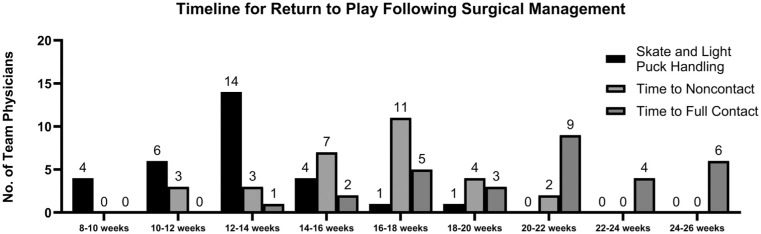
Survey results for return-to-play timeline recommendations of posterior shoulder instability following surgical stabilization.

**Figure 5.**
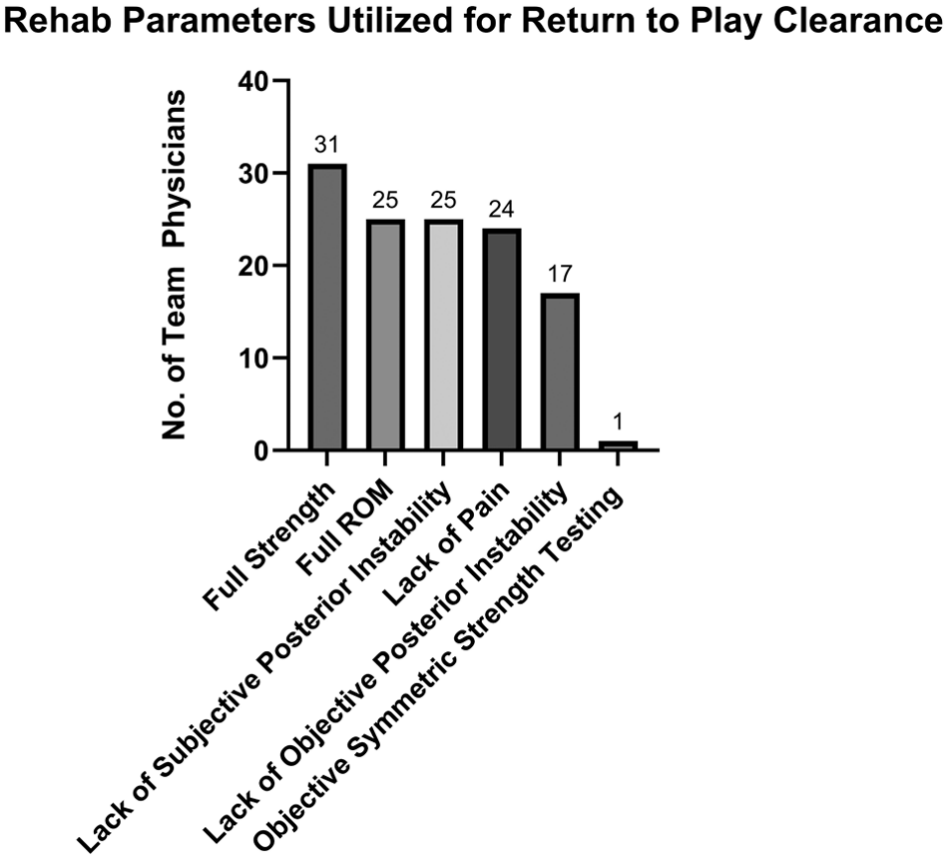
Survey results for important rehabilitation parameters utilized in return-to-play clearance following a posterior shoulder instability event.

Decisions surrounding RTP also varied among physicians. For nonoperative management, most physicians allowed return to noncontact participation in 2 to 4 weeks (18 respondents; 58%) and return to full contact in 4 to 6 weeks (15 respondents; 48%). The full breakdown of responses is presented in Figure 3. Following operative management, skating and light puck-handling drills were most commonly resumed at 12 to 14 weeks (14/30 respondents; 47%). Noncontact return was most commonly permitted at 16 to 18 weeks (11/30 respondents; 37%), and time to full contact was variable, but most commonly allowed at 20 to 22 weeks (9 respondents; 30%). The full breakdown of responses is presented in Figure 4. Across both treatment strategies, functional clearance criteria—rather than fixed timelines—guided RTP decisions. The most common criteria for RTP included full strength of the affected shoulder (31 respondents; 100%), full range of motion (25 respondents; 81%), absence of subjective instability (25 respondents; 81%), and absence of pain (24 respondents; 77%). Important to note that only 1 respondent routinely used objective strength testing compared with the contralateral side for RTP. The other responses are presented in Figure 5.

Routine shoulder bracing during play was recommended postoperatively by only 4 respondents (13%), while 13 respondents (42%) recommended routine bracing in the setting of nonoperative care. The player's position (center, left wing, right wing, defense, goalie) did not affect the decision to recommend a brace in 28 respondents (90%). One respondent was more likely to recommend bracing for centermen, as they take faceoffs. Another respondent was more likely to recommend a brace in goalies. A final respondent was more likely to recommend a brace in defensemen. The involved shoulder being the “top hand” or “bottom hand” on the hockey stick (right-handed versus left-handed shot) was generally not a major factor in treatment decisions, with 28 physicians (90%) indicating it had no influence on management. Three physicians (10%) considered it relevant. One felt that they would be more likely to operate on a bottom-handed injury due to shoulder rotation while shooting the puck. The other 2 physicians felt they would be more likely to operate on a top-handed injury—one explained that the top hand is usually the shoulder initiating contact, and the other explained that the top hand is at higher risk as a result of involvement in poke checking. Most surgeons reported performing arthroscopic stabilization procedures in the lateral decubitus position (21/30 respondents; 70%), with a minority performing the surgery in the beach-chair position (9 respondents; 30%).

## Discussion

The key findings of our survey of NHL team physicians were as follows: first-time soft tissue injuries in the first half of the season were unanimously treated nonoperatively (31/31 respondents; 100%). This changed in the setting of recurrent injury, as 11 respondents (35%) were likely to recommend in-season surgical management in the absence of bone loss. In the setting of recurrent injury with bone loss of ≥15%, the proportion recommending in-season surgery increased to 23 respondents (74%). Most physicians indicated that timing of the injury during the season would not influence their decision regarding management. In the setting of recurrent instability without bone loss, only 8 respondents (26%) felt they would be more likely to attempt nonoperative management and RTP if the injury occurred in the second half of the season with the team in playoff contention. Similarly, 6 respondents (19%) felt they would be more likely to attempt non-operative management and RTP if the injury occurred in the playoffs. These numbers were similar in the presence of recurrent instability with bone loss. There was heterogeneity in terms of offseason management, as 12 respondents (39%) would recommend offseason surgery for a first-time instability event, while 15 respondents (48%) would recommend offseason surgery for a recurrent instability event in the absence of bone loss. In total, 84% of physicians felt that bone loss would influence their decision toward recommending offseason surgery. RTP timelines were variable among physicians. For nonoperative management, the highest proportion of physicians allowed return to noncontact in 2 to 4 weeks (18 respondents; 58%) and return to full contact in 4 to 6 weeks (15 respondents; 48%). Following operative management, light puck-handling drills were most commonly resumed at 12 to 14 weeks (14 respondents; 47%). Noncontact return was most commonly permitted at 16 to 18 weeks (11 respondents; 37%), and full contact was most commonly allowed at 20 to 22 weeks (9 respondents; 30%). The most common functional criteria for RTP included full strength of the affected shoulder (31 respondents; 100%), full range of motion (25 respondents; 81%), absence of subjective instability (25 respondents; 81%), and absence of pain (24 respondents; 77%).

These findings reflect evolving practices in the management of posterior shoulder instability in elite hockey players. Despite historical underrecognition, decisions surrounding posterior instability are now becoming stratified by severity, recurrence, and bone loss thresholds. While surgeons exhibit variation in treatment triggers, trends exist regarding the role of bone loss, recurrent instability, and persistent symptoms in influencing surgical management.

Shoulder injuries in ice hockey players make up anywhere between 8.6% and 21.9% of all upper limb injuries.^3-5,9,15^ The high-speed collision nature of hockey places players at particular risk for injury. When an instability event occurs, there are many clinical options when deciding how to manage the in-season athlete. Many factors can be considered, such as symptomatology, number of episodes, presence of bone loss, and RTP timing, to name a few. No consensus exists for the management of posterior shoulder instability in NHL hockey players, making these findings particularly relevant.

In this survey, all NHL team physicians preferred nonoperative treatment for a first-time posterior shoulder instability in-season. However, 39% would consider surgical stabilization in the offseason, particularly in the presence of persistent pain, large or anteriorly extending labral tears, or partial-thickness rotator cuff involvement. This reflects clinical concern for progressive pathology and the need for structural correction when time allows.

Recurrent posterior instability elicited greater variability in responses. Only 35% of team physicians favored in-season surgery for recurrent instability without bone loss, which rose to 74% when ≥15% bone loss was present. Importantly, more than two-thirds of physicians reported that timing within the season (eg, playoff contention) did not influence their management decisions in the setting of recurrent instability, both with and without bone loss. For those who were influenced by timing, most preferred attempted nonoperative care in playoff situations, prioritizing short-term availability. In the offseason, 48% of respondents would recommend surgery in the setting of recurrent instability, with decisions commonly influenced by anticipated recurrence risk, bone loss, player preference, and the potential for tear progression.

The top reasons for in-season surgical intervention were bone loss, same-season recurrence, persistent pain, subjective instability, and decreased performance—all of which align with broader data showing that these factors predict poor nonoperative outcomes and lower RTP rates.^8,10,16^

Surgical techniques varied substantially, especially when glenoid bone loss was present. For isolated soft tissue pathology, 100% of respondents favored arthroscopic stabilization, consistent with successful outcomes in the literature.^2,12^ However, in the presence of ≥15% bone loss, only 30% recommended a distal tibial allograft. The remainder preferred continuing with soft tissue repair alone (53%), opted for open repair without bone block augmentation (7%), or stated that their decision would be case dependent (10%). This distribution underscores the lack of agreement about thresholds for bone grafting and appropriate surgical technique in posterior instability, mirroring recent debates in the literature.^6^

RTP criteria also demonstrated divergence among physicians. While most surgeons emphasized function-based milestones—full strength, range of motion, and symptom resolution—specific timelines varied. Return to full contact after operative management generally occurred between 16 and 26 weeks, while nonoperative return to full contact ranged from 2 to 6 weeks depending on severity and symptoms. These findings suggest that rigid timelines are unreliable indicators of true readiness, may heighten the risk of reinjury^7^, and may reflect a growing shift from purely time-based criteria to using functional criteria in RTP.

Importantly, our findings highlight ongoing heterogeneity among experts in multiple key areas: (1) whether or not to recommend shoulder stabilization in the offseason for a first-time injury; (2) timing of surgical intervention for recurrent instability—whether during or after the season; (3) which patient factors should lead to early surgical stabilization; (4) bone loss percentage that justifies performing a bony reconstruction procedure; (5) surgical technique preference in the presence of glenoid bone loss; (6) timing of RTP following operative and nonoperative management.

This variability emphasizes the need for further high-quality, prospective research to establish evidence-based guidelines for surgical decision-making and RTP management of posterior shoulder instability in elite contact athletes.

### Limitations

There are several limitations to our study. First, these were all hypothetical scenarios presented to team physicians. They do not perfectly mirror real-life clinical scenarios that contain more nuanced decision-making. This study did not take into account informed consent of the athlete in a shared decision-making model. Second, this is a study based on expert opinion from a single sport and does not contain clinical data. Third, the decisions portrayed by this study have a narrow scope. These scenarios were solely based on NHL hockey players and may not apply to other levels of competition.

## Conclusion

NHL team physicians strongly favor nonoperative management in-season for initial posterior instability events of the shoulder. Recommendation of in-season surgery becomes more likely in the presence of injury recurrence or bone loss, although heterogeneity exists among experts. Injury timing during the season rarely alters treatment decisions, and arthroscopic repair remains the standard approach. Bone block procedures were preferred by a minority of physicians in the setting of bone loss, and there was heterogeneity among surgeons regarding amount of combined bone loss that would lead to the recommendation of a bony procedure. Heterogeneity exists among experts regarding RTP, suggesting a nuanced approach combining both function-based milestones and time.
